# Cross-Jurisdictional Resource Sharing in Local Health Departments: Implications for Services, Quality, and Cost

**DOI:** 10.3389/fpubh.2018.00115

**Published:** 2018-04-26

**Authors:** Debbie L. Humphries, Justeen Hyde, Ethan Hahn, Adam Atherly, Elaine O’Keefe, Geoffrey Wilkinson, Seth Eckhouse, Steve Huleatt, Samuel Wong, Jennifer Kertanis

**Affiliations:** ^1^Yale School of Public Health, New Haven, CT, United States; ^2^Center for Healthcare Organization and Implementation Research, U.S. Department of Veterans Affairs, Bedford, MA, United States; ^3^Colorado School of Public Health, University of Colorado Anschutz Medical Campus, Aurora, IL, United States; ^4^Center for Health Services Research, Larner College of Medicine, University of Vermont, Burlington, VT, United States; ^5^Boston University School of Social Work, Boston, MA, United States; ^6^Boston University School of Public Health, Boston, MA, United States; ^7^West Hartford—Bloomfield Health District, Bloomfield, CT, United States; ^8^Framingham Health Department, Framingham, MA, United States; ^9^Farmington Valley Health District, Farmington, CT, United States

**Keywords:** resource sharing, obesity prevention, physical activity promotion, healthy food activities, food safety, local public health, public health administration models, politics

## Abstract

**Background:**

Forty one percent of local health departments in the U.S. serve jurisdictions with populations of 25,000 or less. Researchers, policymakers, and advocates have long questioned how to strengthen public health systems in smaller municipalities. Cross-jurisdictional sharing may increase quality of service, access to resources, and efficiency of resource use.

**Objective:**

To characterize perceived strengths and challenges of independent and comprehensive sharing approaches, and to assess cost, quality, and breadth of services provided by independent and sharing health departments in Connecticut (CT) and Massachusetts (MA).

**Methods:**

We interviewed local health directors or their designees from 15 comprehensive resource-sharing jurisdictions and 54 single-municipality jurisdictions in CT and MA using a semi-structured interview. Quantitative data were drawn from closed-ended questions in the semi-structured interviews; municipal demographic data were drawn from the American Community Survey and other public sources. Qualitative data were drawn from open-ended questions in the semi-structured interviews.

**Results:**

The findings from this multistate study highlight advantages and disadvantages of two common public health service delivery models – independent and shared. Shared service jurisdictions provided more community health programs and services, and invested significantly more ($120 per thousand (1K) population vs. $69.5/1K population) on healthy food access activities. Sharing departments had more indicators of higher quality food safety inspections (FSIs), and there was a non-linear relationship between cost per FSI and number of FSI. Minimum cost per FSI was reached above the total number of FSI conducted by all but four of the jurisdictions sampled. Independent jurisdictions perceived their governing bodies to have greater understanding of the roles and responsibilities of local public health, while shared service jurisdictions had fewer staff per 1,000 population.

**Implications:**

There are trade-offs with sharing and remaining independent. Independent health departments serving small jurisdictions have limited resources but strong local knowledge. Multi-municipality departments have more resources but require more time and investment in governance and decision-making. When making decisions about the right service delivery model for a given municipality, careful consideration should be given to local culture and values. Some economies of scale may be achieved through resource sharing for municipalities <25,000 population.

## Introduction

Local public health services aim to protect and improve public health. The delivery of such services in the United States is the primary responsibility of local health departments (LHDs), which vary in size, funding level, structure, governance, and services offered (1). With 41% of health departments in the U.S. serving jurisdictions with populations of 25,000 or less and 61% serving 50,000 or less, researchers, policymakers, and advocates have long questioned how to strengthen public health systems in smaller municipalities. In studies examining local public health services and infrastructure, small and rural health departments have been found to provide few of the 10 Essential Public Health Services and face crucial challenges in accessing resources for local prevention work (2–4). An emphasis on efficiency of service delivery for public health services and the potential for economies of scale has led to increasing support for sharing resources across municipalities. Researchers in New York State in 2002–2003 analyzing a cross-sectional sample of 34 LHDs reported the highest per capita expenditures in the smaller county health departments (5), suggesting the potential for inefficiencies in those LHDs. While standard measures of efficiency of service delivery are lacking for LHDs, variation in size of health department has been found to be a primary source of variation in levels of per capita spending in several studies (6, 7). Cross-jurisdictional sharing is intended to increase quality of services (8), access to resources and efficiency of resource use, although benefits and challenges exist (9, 10).

Access to resources is an important concern for LHDs, and a national study following the recession in the early 1990s found that LHDs serving larger populations or multicounty jurisdictions were more likely to report budget increases than LHDs serving populations <50,000 or city/town jurisdictions (11). However, a recent study of two regional health departments in Nebraska cautions that cross-jurisdictional arrangements may not be sufficient to increase access to financial and other resources, and that inadequate or unstable funding may limit the ability of regional health departments to benefit from leveraging resources (12).

In addition to questions of impact of sharing on cost, quality and breadth of service, better understanding of differences between municipalities that pool resources to deliver public health, and those that remain separate are also of interest (13). A study investigating perspectives on collaboration of local public health officials and county commissioners found that public health officials identified key political barriers to collaboration, including commitment to home rule, loss of local input into public health services and priorities, perceived threats to local elected officials, and lack of collaborative government (14). Two recent studies in Connecticut (CT) addressed choices about funding for public health services and decisions to aggregate with other municipalities into multitown LHDs (15, 16). Both studies emphasized the individual factors that influence the decision to remain autonomous, such as variation in population and income in comparison with other local communities (15, 16).

As the long-term economic outlook for local and state governments remains bleak, an increasing number of governmental and non-governmental policymakers are considering cross-jurisdictional collaboration to meet the needs of citizens in the most efficient manner possible (17). This growing interest is not without challenges for the field. While there is strong evidence of potential cost savings and efficiencies of moving to shared services (18, 19), there is a lack of information about the potential challenges of moving from an independent to a comprehensive shared service delivery model in public health. In addition, although the current model of government, with its emphasis on highly localized services, may be desired by local constituents, the willingness of local tax payers and capacity of governments to fund these services is limited. There is not yet a clear understanding of how cross-jurisdictional sharing arrangements impact implementation of public health services. The 2012 summary of the National Public Health Services and Systems Research Agenda identified a better understanding of how cross-jurisdictional models of resource sharing affect implementation of public health services and how inter-organizational relationships and interactions affect implementation of public health services as priorities under the public health system structure and performance topic area (20).

## Materials and Methods

### Study Context

In 2013, the public health practice-based research networks (PBRNs) in CT and MA began collaborating to address gaps in research on the relative strengths and costs of two service delivery models – the independent model, in which a single-municipality delivers all locally available services to its residents, and the comprehensive shared services—or district—model, in which multiple municipalities deliver a comprehensive set of services to their combined populations. The PBRNs in each state are comprised of local public health practitioners and academics who work together to identify research questions that are important for the field. Each team had between 6 and 8 members. CT and MA share a similar government structure that makes a cross-state comparison possible. Both states have strong home rule authority, which means that local governments are able to set up their own system of governing and local ordinances so long as they are within the bounds of state and federal constitutions. Unlike most other states with strong county governments, the responsible agent for public health services is at the municipal level, resulting in a large number of health departments with wide variation in the scope and approach to service delivery across CT’s 169 and MA’s 351 cities and towns. Over the last few decades, a mix of service delivery models have been developed in both states to provide local public health services, ranging from independent to comprehensive shared services.

Connecticut has a system of single-municipality health departments and fully integrated multi-municipality districts, where governance and resources are shared through a Board of Health. Over the last 35 years 107 CT municipalities have chosen to join a district to provide their local public health services. In MA, the movement toward formal cross-jurisdictional service delivery models is newer, with a few exceptions. Unlike CT, there are several service-sharing models that are currently being implemented, ranging from sharing a single service to delivery of a comprehensive set of public health services under one administrative structure across multiple jurisdictions. In addition, regardless of service delivery model, each municipality in MA has its own board of health that retains local authority.

Drawing on CT’s long-standing experience with cross-jurisdictional service sharing and MA’s more recent experiences, our PBRNs sought to add to the body of knowledge on the structure and organization of local public health services in the United States. We collaborated on a mixed methods study to assess the effects of cross-jurisdictional resource sharing on implementation of public health services, as well as to better understand strengths and challenges of each approach from the perspective of local health directors. Data were collected from July 2015 to February 2016.

With responsibility for local public health services vested in municipal governments and the history of cross-jurisdictional resource-sharing arrangements, CT and MA provide complimentary locations for studying the relationship between service delivery model and the quality, breadth, and cost of local public health services. For this project, we focused on two different service delivery models – independent health departments with local boards of health (MA & CT) and comprehensive shared service delivery under a single administrative structure with either one board of health (CT) or local boards of health for each municipality (MA), to maintain consistency within and between comparison groups in each state.

Given variation in local public health services across municipalities and states, we selected three categories of services as representative of chronic disease prevention (obesity prevention), communicable disease control (enteric disease control), and environmental health protection [food safety inspections (FSIs)]. Categories were chosen based on availability of data and variation in services across municipalities in CT and MA. For example, some data for enteric diseases were available from state databases. Food protection is a statutory requirement and each state requires annual reporting. In both states, obesity prevention is not a mandated service and engagement in prevention activities may differentiate higher capacity LHDs from lower capacity LHDs.

#### Sampling

Connecticut has 21 multi-municipality districts, which include 107 towns and serve a total population of approximately 1,715,000. CT districts include between 2 and 20 municipalities. MA has 7 comprehensive, multi-municipality public health districts, which include a total of 38 municipalities and serve a total population of approximately 529,000. In order to recruit a mix of comprehensive resource-sharing municipalities and non-sharing or independent municipalities that are similar in terms of population, we utilized a multistage stratified random sampling approach. *Stage 1*: In CT, we created three strata of districts based on number of member municipalities (>10, 5–9, and <5). Within each strata, we randomly selected districts to recruit, and recruited until we obtained districts representing at least 30 member municipalities. For each selected district, all member municipalities were included in the sample. In MA, all 38 municipalities involved in comprehensive multi-jurisdictional resource-sharing arrangements were recruited for participation. *Stage 2*: After selection of districts (CT) and resource-sharing municipalities (MA), the populations of each municipality in the selected jurisdictions were categorized into four strata (<10,000; 10–25,000; 25–50,000 and >50,000) in order to select single-municipality health departments with comparable populations. The total number of towns in each strata for all selected multiple municipality jurisdictions became the target number of single-municipality health departments that were randomly selected for recruitment from the same population strata and geographic characteristics. If there were not a sufficient number of towns in a stratum, then towns of the next closest strata were used.

After conducting the multistage stratified random sampling, we created a sample frame of approximately 69 municipalities from regional service-sharing LHDs and 69 similarly sized single-municipality LHDs, for a total of 138 participating municipalities. Initial invitations to participate in the study were sent *via* email, and lead PBRN partners conducted follow-up telephone calls, to build on the relationships within each PBRN. Health directors that agreed to participate were entered into a raffle to win an iPad following completion of the data collection.

We contacted 17 sharing departments and 15 agreed to participate (88%). We contacted 113 independent departments and 54 agreed to participate (48%). CT recruited 100% of their shared service municipalities to participate while achieving 70% response rate for non-shared service municipalities. CT’s total response rate was 86%. MA acquired 80% response rate for their shared service municipalities, and 31% for non-shared service municipalities. MA’s total response rate was 49%. The final sample included 54 independent departments (22 in CT, 32 in MA) and 15 sharing departments (8 in CT representing 38 municipalities, and 7 in MA representing 38 municipalities). Eighty percent of participating municipalities had populations <25,000.

### Quantitative and Qualitative Data Sources

Quantitative data were drawn from existing data and semi-structured interviews with LHD officials; qualitative data were drawn from semi-structured interviews.

#### Existing Data

We created a unified database of community demographic and economic variables including population, percent of the population below the poverty line, and the unemployment rate using the 2013 American Community Survey. The Connecticut Department of Public Health (DPH) provided municipal level data on reportable enteric infections for the relevant time period. Similar data were not available for MA, so enteric disease results are only reported for CT. CT has a Centers for Disease Control-funded Emerging Infections Program (EIP) that conducts much of the follow up and investigations for enteric infections in CT. The CT DPH provided staffing cost estimates for their staff’s work on enteric investigations.

#### Semi-Structured Interviews: Local Health Directors

To be able to accurately capture indicators of breadth, quality and cost efficiency of service implementation and dissemination, a semi-structured interview was developed that included both quantitative and qualitative questions. The quantitative, closed-ended questions covered four domains: (1) organizational characteristics, (2) perceptions of political priorities and motivations (14), (3) governance structure, and (4) measures of breadth, cost and quality in the service areas of food inspection, enteric disease and obesity prevention. The latter were drawn from the MPROVE Measures, a standard set of measures developed with support from the Robert Wood Johnson Foundation to increase consistency in data regarding the reach, volume, capacity, quality, and cost of core public health services (10). These measures cover three domains: chronic disease prevention, communicable disease control, and environmental health protection, with bundles of indicators for 33 different service areas. We utilized one bundle of services from each of the three domains of the MPROVE measures related to our services of focus. Variables collected are described in Table S1 in Supplementary Material. In both states, obesity prevention is not a mandated service and was included to determine whether engagement in prevention activities varied between sharing and independent LHDs. Questions on obesity prevention are shown in Table S2 in Supplementary Material.

The semi-structured interviews also included open-ended questions to gain insights into (a) perceptions of the strengths and challenges of each participants’ service delivery model, (b) descriptions of the process to develop and approve local public health budgets, (c) clarification on what is included in local public health budgets, and (d) explanation of quality indicators (e.g., details about how retail food inspection staff are supervised, what the evaluation of their retail food inspection program entails, if indicated that there is one). Given the potential for local variability in how public health departments operate, interviewers were instructed to probe for clarification in responses to open-ended and close-ended questions as needed.

Health directors working in single jurisdictions were asked to provide responses for their municipality only and health directors serving multiple jurisdictions responded to questions for each municipality within their service-sharing arrangement. After scheduling an interview, the interviewing team e-mailed participating Health Directors a document outlining pertinent information to bring to the interview. Such information included previous year budgetary documents, staff salaries and time spent working in key services of focus. Health directors were encouraged to use these documents to answer the closed-ended questions.

##### Quantitative Analysis

Excel spreadsheets were converted into Stata data files (Stata 14, Stata Corp) for analysis. Means and 95% CI are reported for continuous variables, and comparisons between independent and sharing departments were made with non-parametric tests (Wilcoxon rank sum) due to the skewed nature of the data. Categorical variables were compared using a chi-squared test. Logistic regression was used to identify demographic and organizational variables associated with sharing LHDs.

##### Cost Analysis

While questions covered full costs of providing services, including indirect rates and overhead costs, few health departments were able to provide this information. In particular, single-municipality health departments were generally integrated into the city budget, with staff benefits, space and utilities covered elsewhere in the city’s budget. To be able to utilize comparable numbers for sharing and independent departments, we focused on staff costs. We calculated the staff costs of enteric diseases in CT and food inspections in MA and CT health districts and health departments per 1,000 population, and compared the costs among those that are part of a health district vs. those that are an independent LHD. We used general linear models, stratified by state to test the hypotheses that participating in jurisdictional-sharing arrangements affects staffing cost of food inspections and enteric case investigations after controlling for population, full-time/part-time status in CT, number of municipalities in service-sharing arrangements, governance structure, and socioeconomic status as measured by% of population below poverty level. A quadratic term (number of establishments^2^) was added to generate the curve showing the relationship between total cost of inspections and number of inspections.

##### Qualitative Analysis

All semi-structured interviews with local health directors were audio-recorded and transcribed. Transcripts from the interviews provided an opportunity to accurately record responses to open-ended questions interspersed throughout the semi-structured interview. Transcripts also provided the research team with the ability to examine variation across states and service delivery models with respect to how close-ended questions were interpreted and answered.

A subgroup of the larger research team worked together to organize and analyze the qualitative data. All qualitative data were uploaded into Dedoose, a qualitative data software that supports team-based coding and analysis. The first step was to organize the large volume of data by creating a coding scheme consisting of high level codes that corresponded to question type. This allowed the team to easily pull text from interview segments related to specific questions or topics. The team then prioritized segments of the semi-structured interviews for additional thematic analysis. These included all open-ended questions related to the strengths and challenges of the service delivery model, information about the local public health budgeting process, and all quality measures for each of the three public health services. Thematic codes were initially developed by reviewing a subsample of 10 interviews (five from each state and mix of service delivery models) and then identifying and labeling types of responses to open-ended questions. As additional waves of data were coded, adjustments to the thematic codes for select questions were made by the analysis team. Once all the data were coded, the team reviewed data associated with select codes (e.g., strengths and challenges of service delivery model, details about quality indicators). The analysis entailed summarizing coded data, looking for similarities and differences by state and service delivery model. Results of these analyses were placed in tables and shared with the larger research team for discussion.

##### Ethical Review

The Yale University Human Subjects Committee and Cambridge Health Alliance Institutional Review Board reviewed this research and found it to be exempt from IRB review under federal regulation 45 CFR 46.101(b)(2).

## Results

### Municipality Characteristics

Table 1 provides an overview of key municipal characteristics by service-sharing model. Independent LHDs had lower population density, and were also significantly more likely to have an elected chief executive. Poverty rate, rural/urban, unemployment and race and ethnicity did not vary between sharing and independent jurisdictions. Municipal budgets and public health budgets per 1,000 population were similar (*p* = 0.60) across independent and sharing LHDs. Few larger municipalities (>50,000 population) choose to participate in comprehensive sharing arrangements, so the sample is primarily made up of smaller municipalities.

**Table 1:** Characteristics of independent and sharing municipalities.

	Resource sharing (*n* = 76)	Independent (*n* = 54)	
**Municipality population, % (n)^a^**			*p*-Value^b^
<5,000	27.6% (21)	16.7% (9)	0.176
5,000–10,000	18.4% (14)	37.0% (20)	
10,000–25,000	34.2% (26)	17.8% (15)	
25,000–50,000	17.1% (13)	16.7% (9)	
>50,000	2.6% (2)	1.9% (1)	
**Municipality type, % (*n*)**							
Rural	47.4% (36)	53.7% (29)	0.168
Suburban	15.8% (12)	24.1% (13)	
Urban	36.8% (28)	22.2% (12)	
**Legislative structure, % (*n*)^b^**
Elected council	46% (23)	37.8% (14)	0.403
Open town meeting	60% (50)	40% (34)	
Representative town meeting	33.3% (2)	66.7% (4)	
**Executive structure, % (*n*)^b^**
Mayor (elected)	40.4% (19)	59.6% (28)	0.01
Manager (appointed)	66.7% (52)	33.3% (26)	
Other	100% (5)	0%	
**Demographics, mean (SD)**	(*n* = 15)	(*n* = 54)	
Poverty rate	5.76 (0.89)	5.32 (0.66)	0.79
Unemployment	7.17 (0.35)	7.61 (0.35)	0.52
Population	15,586 (22,637)	14,729 (12,240)	0.8
Pop per sq mile	937 (270)	615 (60)	0.08
Municipal budget per 1,000 population	2.92M (240,400)	3.25M (377,403)	0.6
Public Health budget per 1,000 population	15,170 (1,630)	16,340 (1,800)	0.74
**Race and ethnicity, mean% (SD)**
Black	3.8% (1.2)	5.9% (3.7)	0.59
Hispanic	5.6% (0.011)	4.4% (0.55)	0.31

*^a^Proportions are with respect to the total number of sharing or independent municipalities in that size range in both Connecticut and Massachusetts*.

*^b^Wilcoxon Rank Sum Test*.

### Perspectives on Strengths and Challenges of Sharing and Independent Approaches

Health directors leading independent and shared service public health departments had different perspectives regarding the strengths and challenges of their models. Tables 2 and 3 provide an overview of the most common themes that emerged from health directors working under different models. These qualitative themes were generally supported by the quantitative data related to political support, breadth and quality of services delivered.

**Table 2:** Perceived strengths and challenges of independent health department models.

Description of strengths of model	Illustrative examples
Deep knowledge of local community	“I think basically that we deliver services *with* the community we are working for… We are community based, so we have to be very aware what your issues are and address them…. But you got to know that population well in order to do it.” (CT)
Ability to be responsive to stakeholders within municipality	“I think it’s both accessibility and direct conversation…we are small, we are easily accessible.” (CT)
Infrastructure to support interoperability across municipal departments	“I think the biggest strength is knowing the community so well that we work very closely with building inspector, the plumbing and electrical; they are on the same department. I can just walk down the hall and we can talk about a building…That’s really helpful.” (MA)
Freedom to make decisions for community without getting “bogged down” in bigger decision-making processes	“As being a standalone, we’re able to make decisions without having to involve too many people so when we need to make these major decisions nothing gets bogged down… ”(MA)

**Description of challenges with model**	

Limited capacity to consistently fulfill state mandated responsibilities	“We have far too many responsibilities and this office is way understaffed to really do an exemplary job on all of our mandates. There are some state mandates that we almost never get to unless there is a crisis and there are other mandates that we do a moderate job. But because there is only one full time person and a part time person…some things are given short shrift.” (MA)
Limited resources (human and financial) to provide services outside of mandated public health services	“As far as doing community health programs we do lack resources to provide big programs to the town. We have a substance abuse, prescription drug problem here. When I first came on board the police had said [X town] is the worst for heroin… But me being a one man show having to go out and do all the state mandated inspections, it is a little difficult to tackle programs for the residents in the community on my own.” (MA)
Hiring and retaining a qualified workforce with diverse experience and training	The biggest challenge is expanding our scope of services based on limited financial resources… we do cross training here, we have people that wear several hats. But it’s a challenge because we are just very limited in our ability to retain staff based on our budgeting and to be able to expand our service model (CT)
Working in isolation to protect and promote public health	“I think it can be lonely because you one doesn’t have a lot of colleagues in a smaller health department to bounce things off” (MA)

**Table 3:** Perceived strengths and challenges of comprehensive shared service models.

Description of strengths of model	Illustrative examples
Capacity to hire and retain staff with diverse expertise and experience	We now have a community health coordinator who is also trained as an RN. We have an emergency preparedness coordinator and we have staff like me that have expertise in communicable disease and chronic disease prevention. Any one of those of towns independently could never afford to staff up a health department with that diverse of staffing (CT)
Capacity to offer public health services beyond regulatory mandates	Our strengths is that we’re providing more than just environmental health… The [towns in our region] are getting the full spectrum of public health services that they normally would not have on a regular basis (MA)
Flexibility in financing and programming to adapt to changing needs locally, regionally, and nationally	Where a lot of times within a municipal department, if they make money off a service, it goes back into the general fund and it doesn’t necessarily increase the types or volumes of services that they are allowed to do. So being a district, we have a lot more flexibility to be able to use it in a way that we see fit… If we see an opportunity, we can go after it (CT)
Consistency in rules, regulations, and regulatory processes across neighboring towns	Consistency. I don’t know how big an advantage that is to the individual town, but to the residents and the business owners it’s a big plus, because then the amount of energy required to do business in all of our towns is the same vs. what was in place before – figuring out who you have to call, where to get your permit, who you are going to pay and how you pay it (MA)

**Description of challenges with model**	

Balancing good customer service with efficiency in service delivery	I would say a challenge, it’s not so much our model but the rural nature of our district, is it’s just a challenge geographically driving… I mean that comes down to efficiency but you have to balance out against responsiveness and satisfaction just as well (MA)
Complexity of maintaining good working relationships with diverse stakeholders and their communities	We serve six municipalities, so we serve six elected officials, six building inspectors and six social agencies. There is a huge volume of personnel that we deal with which is very distinct from a health department serving one municipality (CT)
Working regionally with municipalities that think and plan locally	Towns don’t think regionally. So while we are a district health department, when it comes to doing a community needs assessment or a health improvement plan or engaging the community, they don’t think as the [name of district]. They think of themselves as [X town], as [Y town], as [Z town]. So that’s a big struggle… (CT)

Directors serving independent health departments highlighted a deep knowledge of their communities and the ability to quickly respond to constituents’ questions, interests, and needs. Many also highlighted strong working relationships across municipal departments, allowing for greater cooperation and interoperability on complex issues, such as hoarding cases or other building-related complaints. However, many expressed challenges related to hiring and retaining staff with diverse expertise and skills. This was particularly true in smaller jurisdictions (populations 25,000 or less), where the health directors often considered themselves to be a “one person show.” These participants highlighted challenges with consistently meeting respective state mandates for inspectional services and communicable disease control and rarely had time to plan for any additional essential public health services (e.g., health surveillance, mobilization of community partnerships, and evaluating services).

Health directors leading comprehensive shared service departments noted a number of common strengths of their model. For those serving smaller jurisdictions, they noted an increased capacity to hire well trained staff from diverse educational backgrounds. Sharing resources with other jurisdictions allowed them to offer a broader range of community health and prevention programs beyond those mandated by the state. Working collaboratively also opened up opportunities for diversifying funding through grants and other revenues, allowing flexibility in programming to meet the changing needs of collaborating communities. Other strengths included consistency in regulatory practices and code enforcement across municipalities. However, these health directors reported challenges with balancing responsiveness to local needs with efficiency in service delivery. The geographic spread of municipalities within some shared service departments exacerbated this challenge. In CT, the comprehensive shared service departments are referred to as districts and exist independent of any one municipality. Their responsiveness was challenged by feeling like an “outsider” to the forums where local policies and decisions are made. Disparate political views and values among collaborating municipalities were consistent challenges in both states, requiring time and energy for consensus-building related to the design and delivery of programs and services.

### Demographic and Organizational Factors Associated With Service Sharing

Multivariate analysis of variables associated with increased likelihood of being in a resource-sharing model identified the type of chief executive, type of legislative body, population strata and poverty. Municipalities with appointed executives had 20 times higher odds of being in a resource-sharing model than municipalities with elected executives (OR = 20.7; *p* = 0.001) (Table 4).

**Table 4:** Demographic and organizational characteristics associated with service sharing.

	Odds ratio	*p*-Value	Adjusted odds ratio^a^	*p*-Value
**Municipality type (rural is reference)**				
Suburban municipality	0.74 (0.29, 1.87)	0.53	0.52 (0.30, 8.9)	0.65
Urban municipality	1.88 (0.82, 4.33)	0.14	5.1 (0.22, 118)	0.31
**Legislative structure (elected council is reference)**				
Open town meeting	0.91 (0.41, 2.02)	0.82	0.29 (0.04, 1.9)	0.19
Representative town meeting	0.304 (0.49, 1.88)	0.20	0.11 (0.004, 2.9)	0.19
**Executive type (elected mayor is reference)**				
Appointed manager	4.43 (1.4, 13.7)	0.01	20.7 (3.4, 125.5)	<0.005

*^a^Adjusted for Municipality type, legislative structure, executive type, population strata, and poverty rate*.

### LHD and Local Government Relations

Fifty-nine percent of single-municipality respondents reported meeting more than 10 times per year with the chief executive, compared with only 33% in multiple municipality jurisdictions (Table 5). Perceived understanding of public health by the chief executive and BOH members did not vary significantly by sharing or non-sharing jurisdictions, although perceived understanding of town council and finance committee members were higher in non-sharing jurisdictions (Table 5). Resource-sharing jurisdictions reported significantly more BOH members, particularly in CT, which was likely due to the need to have representation from all member municipalities (Table 5).

**Table 5:** Board of health and government relations.

	Sharing (*n* = 15)			Independent (*n* = 54)			*p*-Value
**Board of health**							
No BOH rep	0			18 (33%)			<0.05
Appointed BOH	8 (53.3%)			14 (26%)			
Elected BOH	4 (27%)			22 (41%)			
Average BOH members	15.1 (3.1)			2.5 (0.28)			<0.05

**Frequency of meetings^a^**	**<4/year**	**4–9/year**	**>10/year**	**<4/year**	**4–9/year**	**>10/year**	

Chief executive	20% (3)	46.7% (7)	33.3% (5)	33.3% (18)	7.4% (4)	59.3% (32)	<0.05
Alderman	73.3% (11)	26.7% (4)	0.0%	74.1% (40)	16.7% (9)	9.3% (5)	0.37
Finance committee	93.3% (14)	6.7% (1)	0.0%	90.7% (49)	3.7% (2)	5.6% (3)	0.58
Board of health	6.7% (1)	13.3% (2)	80% (12)	38.9% (21)	7.4% (4)	53.7% (29)	0.06

**Decision-making authority^c^**	**BOH**	**Chief executive^b^**	**Other**	**BOH**	**Chief executive^b^**	**Other**	

Fire health director	73.3% (11)	6.7% (1)	0.0%	35.2% (19)	24.1% (13)	24.1% (13)	<0.05
Hire health director	73.3% (11)	6.7% (1)	0.0%	35.2% (19)	29.6% (16)	20.4% (11)	<0.05
Set fines	73.3% (11)	6.7% (1)	0.0%	50% (27)	5.6% (3)	35.2% (19)	<0.05
Approve public health regulations	93.3% (14)	0.0%	0.0%	57.4% (31)	7.4% (4)	35.2% (19)	<0.05

	**Sharing (***n*** = 77)**			**Independent (***n*** = 54)**			
**Understanding of public health**	**Excellent or good**	**Fair or poor**	**Don’t know**	**Excellent or good**	**Fair or poor**	**Don’t know**	

Chief executive	72% (54)	28% (21)	0	78% (39)	20% (10)	2% (1)	0.30
Alderman	30% (22)	49% (36)	22% (16)	40% (21)	60% (31)	0	<0.05
Finance committee	10% (7)	65% (47)	25% (18)	33% (17)	51% (26)	16% (8)	<0.05
Board of health	95% (71)	5% (4)	0	89% (32)	8% (3)	3% (1)	0.28

*^a^Frequency of meetings is not available at the municipal level for multi-municipality health departments*.

*^b^Chief Executive includes elected mayors and appointed town managers*.

*^c^Percentages do not sum to 100% due to missing data, where respondents could not answer the questions*.

### Impacts on Implementation: Staffing and Services

Independent LHDs had a higher FTE/1,000 population (0.22 vs. 0.14; *p* = 0.07; Table 6). Almost all sharing LHDs (93%) had a director with graduate level public health training (MPH), whereas directors of independent LHDs were more likely to have a bachelor’s degree or an MD or PhD (Table 6). The MD/PhD finding is driven by smaller health departments; in CT, smaller health departments are more likely to hire a part time director with an MD or PhD and in MA small health departments may be run by a volunteer board of health with a chairperson that holds a MD/PhD. With respect to public health services, those that are mandated by the state were more likely to be provided in both service delivery models. Most core public health services were offered at similar rates by both service delivery models. Core public health services that varied by sharing status are shown in Table 6. There were some notable differences between the states, with sharing health departments in CT less likely to offer public health nursing in comparison to independent departments (34% vs. 73%). This was not the case in MA, which showed slightly more sharing health departments offering public health nursing services than independent models (82% vs. 75%).

**Table 6:** Services and staffing of independent and sharing health departments.

	Sharing (*n* = 15)^a^	Independent (*n* = 54)^a^	
**Staffing**			*****p***-Value^b^**
FTE/1,000 population	0.14	0.22	0.07
Director has MPH	93.30%	50%	
Director has bachelor’s degree	6.70%	33.30%	
Director has MD or PhD	0	16.70%	
**Core public health services that varied by service delivery model^c^**			
Animal control	74%	93%	0.07
Lead inspections	97%	81%	0.004
Mosquito control	67%	39%	0.002
Natural bathing water testing	87%	70%	0.02
Nail salon inspections	82%	65%	0.03
Public health nursing (CT specific)	58%	74%	0.06
Public pool inspections	99%	85%	0.004
**Any obesity prevention initiatives, % (***n***)**			
Healthy food initiatives	93.3% (14)	50.0% (27)	0.002
Physical activity initiatives	86.7% (13)	46.3% (25)	0.005

*^a^Proportions are with respect to the total number of sharing or independent municipalities*.

*^b^Wilcoxon Rank Sum Test*.

*^c^Core public health services that did not vary by service delivery model include: communicable disease surveillance and investigation, emergency preparedness, enforcement of tobacco control regulations, food-related inspections, flu vaccinations, housing complaints, inspections of body art businesses, nuisance complaints, public medical waste disposal, record keeping (death certificates, services rendered, etc.), routine vaccinations, septic system inspections, and water supply well permitting*.

### Impacts on Implementation: Obesity Prevention Activities

**Figure 1:**
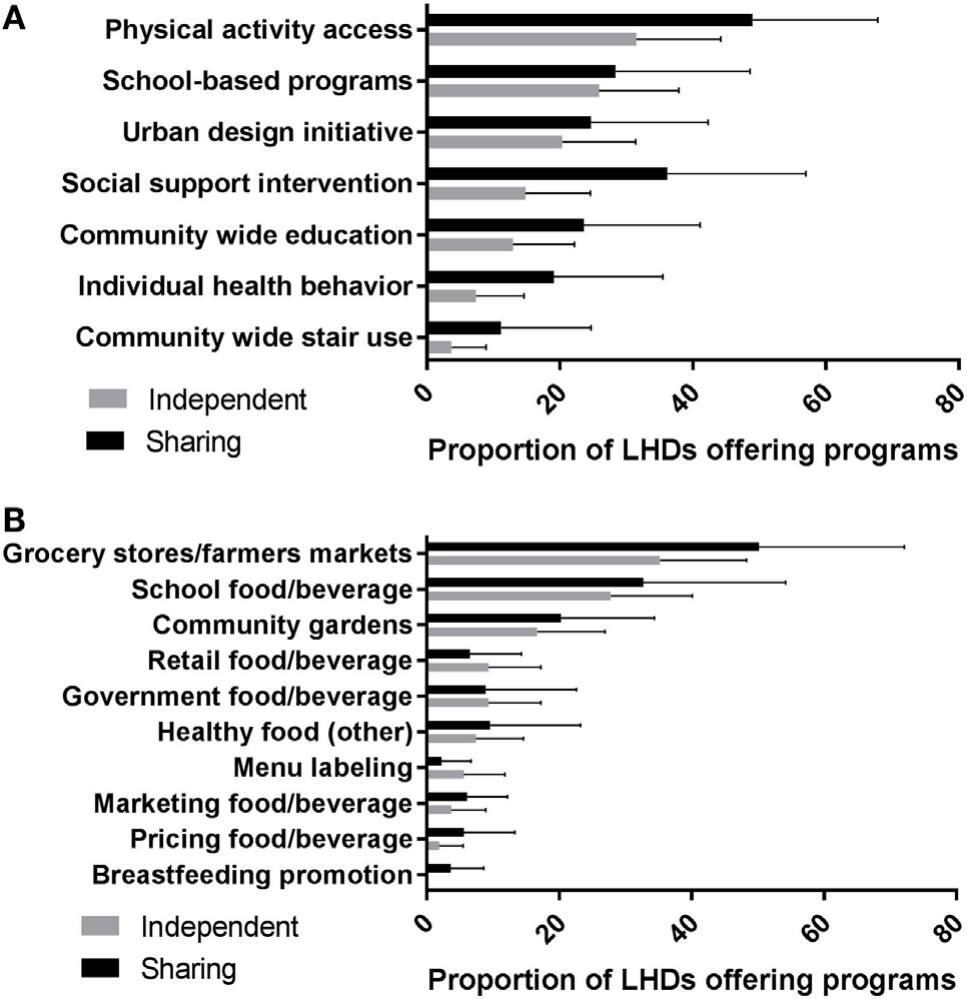
The proportion of communities were each approach to **(A)** physical activity interventions and **(B)** healthy food initiatives are available. Independent and resource-sharing values are for the proportion of the total number of participating municipalities were such activities are ongoing.

Almost all (93.3%) resource sharing departments reported participation in community level healthy food initiatives, and the vast majority (86.7%) reported participating in physical activity promotion initiatives (Table 6). Participation in obesity prevention activities among independent departments was lower, with almost half reporting participating in physical activity initiatives and healthy food activities (Table 6). Physical activity promotion initiatives included stair use, school programs, urban design initiatives and community wide education approaches. The most common physical activity initiative reported was to improve access to physical activity locations (Table S2 in Supplementary Material), with 31.5% of independent and 48.9% of sharing departments reporting such initiatives (Figure 1A). Grocery store and school-based healthy food initiatives (Table S2 in Supplementary Material) were the most common healthy food initiatives reported by both independent (35.2%) and sharing (50.2%) departments (Figure 1B), and school food initiatives were next most common among both kinds of departments.

### Impacts on Implementation: Food Safety Activities

**Figure 2:**
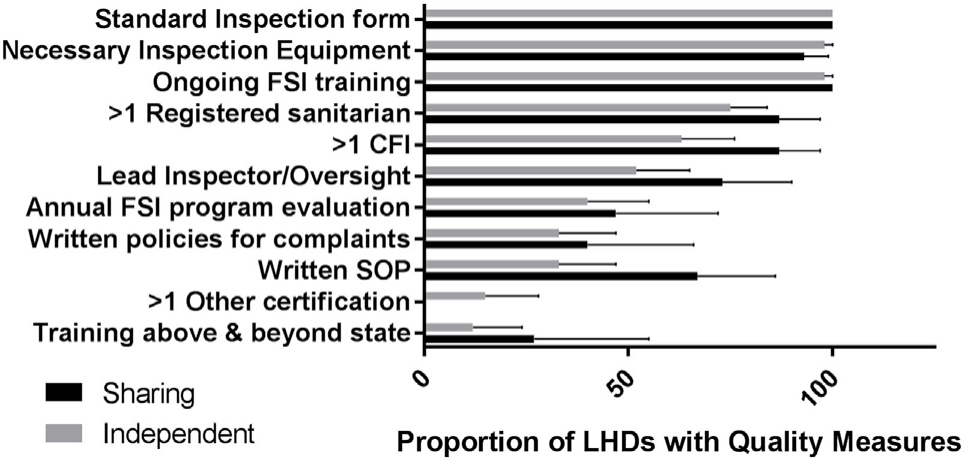
Proportion of local health departments with each food safety inspection (FSI) quality indicator. Quality indicators include (1) availability of certified FSI personnel, (2) ongoing FSI training, (3) on going FSI training that is above and beyond state requirements, (4) availability of a standard inspection reporting form, (5) written standard operating procedures, (6) presence of formal program oversight, (7) availability of written policies for complaints, (8) availability of necessary inspection equipment, and (9) an annual FSI program evaluation.

There were significantly more food service establishments, and significantly more retail food inspections conducted in sharing departments in comparison with independent departments (Table 7). However, after adjusting for population, there was no difference in the number of food service establishments or retail inspections between independent and sharing departments. Sharing departments were more likely to have five or more of seven food service quality indicators^1^ assessed (73% vs. 46%; *p* = 0.064) (Figure 2). The most common quality indicators across both models were formally trained food safety inspectors, use of a standard inspection reporting form, and availability of equipment needed for food inspections. Both states require at least minimal training for retail food inspectors, although CT requires more in-depth and ongoing training in comparison to MA. Nearly all respondents reported using their state’s inspection form, and that equipment for food inspections was not a challenge. Written standard operating procedures and procedures for responding to complaints were not commonly reported, but those who did were likely to report working toward or had achieved public health accreditation or enrollment in the FDA’s Voluntary Retail Food Safety Program. Having a designated supervisor to oversee the inspectional service was more likely among shared service departments and independent health departments in urban or suburban communities. Smaller health departments often noted their program was too small to have a supervisor, as only one person was usually responsible for handling food inspections. The weekly staff hours devoted to food safety per 100 food establishments was very similar between sharing and independent departments (18.1 vs. 22.2; *p* = 0.86).

**Table 7:** Food safety and enteric disease activities.

	Resource sharing (*n* = 15)	Independent (*n* = 54)	*p*-Value
**Food protection**			
No. of food service establishments/licensed food vendors, mean (95% CI)	401.5 (148, 654)	91.4 (68, 115)	0.002
No. of retail food inspections conducted during the past 12 months, mean (95% CI)	679 (246, 1112)	182 (126, 238)	0.0004
No. of food service establishments/licensed food vendors per 1K population, mean (95% CI)	0.135 (0.040, 0.055)	0.249 (0.186, 0.312)	0.02
No. of retail food inspections conducted during the past 12 months per 1K population, mean (95% CI)	8.86 (7.3, 10.4)	10.9 (9.15, 12.6)	0.4
Weekly staff hours devoted to retail food safety inspection, protection and control per 100 retail food establishments, mean (95% CI)	18.1 (12.0, 24.3)	22.2 (16.8, 27.5)	0.86

	**Resource sharing (***n*** = 8)**	**Independent (***n*** = 22)**	*****p***-Value**

**Foodborne/waterborne enteric disease (Connecticut only)**			
No. of cases of enteric disease in past 12 months (per 1K population), mean (95% CI)	0.33 (0.25, 0.41)	0.45 (0.37, 0.53)	0.11
Proportion of investigations lost to follow up, mean (95% CI)	0.02 (0.00, 0.04)	0.09 (−0.01, 0.18)	0.55
LHD defers ED investigations to other agents, *n* (%)	4 (50%)	12 (70.6%)	0.32

### Impacts on Implementation: Enteric Infections in Connecticut

There were slightly more cases of enteric disease in independent departments (0.45 per 1K population vs. 0.33 per 1K; *p* = 0.11; Table 7). Less than 10% of infections are lost to follow up in independent (9%) and sharing (2%) departments (*p* = 0.55; Table 7). Seventy percent of independent departments in CT defer their enteric disease investigations to the state and the CT EIP compared with 50% in resource sharing (*p* = 0.32). This program provides assistance with enteric disease investigations when requested by both sharing and independent departments.

### Impacts on Implementation: Obesity Prevention Costs

Shared service departments invested more staff resources in obesity prevention activities than independent departments. Thus, expenditures on obesity prevention efforts, which are not mandated services in either state, were higher in resource sharing health departments than independent ones ($180.7 vs. $69.5/1,000 population). The difference was statistically significant for healthy food activities (*p* = 0.04; Table 8), but not for physical activities or overall costs in this service area.

**Table 8:** Staffing costs for obesity prevention, enteric disease control, and food service inspection programs.

Staffing costs, mean (95% CI)	Independent	Resource sharing	*p*-Value**
**Obesity prevention staffing costs per 1,000 population (95% CI)^a^**			
Physical activity	46.7 (0.3, 93.0)	136.2 (33.9, 238.5)	0.14
Healthy foods	20.3 (−14.9, 55.4)	120.0 (42.4, 197.6)	0.04
Overall	69.5 (0.9, 138.0)	180.7 (29.3, 332.1)	0.22
**Enteric disease investigation staffing costs^a^**			
Cost per ED investigation	1,352 (685, 2,019)	2,321 (1,006, 3,637)	0.24
ED cost per 1K population	461 (298, 625)	463 (102, 824)	0.99
**Food service inspection costs^a^**			
Cost per food inspection	135.7 (95.8, 175.6)	93.6 (5.4, 181.8)	0.43
Cost per food establishment	155.1 (109.7, 200.4)	123.5 (25.2, 221.8)	0.59
Cost per 1K population	1,468 (1,070, 1,870)	1,018 (128, 1,909)	0.4

*^a^Adjusted for unemployment and square miles*.

**Ordinary least-squares regression.

### Impacts on Implementation: Enteric Infection Costs in Connecticut

Cost per enteric disease investigation was much higher among resource-sharing departments than in independent departments ($2,321 vs. $1,352), although this difference did not reach statistical significance (0.24; Table 8). The cost for enteric disease control per capita in CT was virtually identical in sharing and independent departments (463 vs. 461; *p* = 0.99) (Table 8). Support of the EIP to health departments throughout the state may be an important driver of the similar cost estimates for sharing and independent health departments.

### Impacts on Implementation: Food Safety Inspection Costs

While the total number of FSIs for independent and sharing departments is significantly different, the cost per FSI is not (Table 8). Cost per FSI is primarily driven by the number of inspections conducted. State, status as a resource sharing or independent department, unemployment and indicators of inspection quality were insignificant in the regression model to predict cost. The total cost of inspections increases at a decreasing rate, and the minimum cost per inspection is reached at 804 inspections. Only four participating departments conducted over 804 inspections (one independent and three sharing).

## Discussion

This research study was one of the first to examine, across multiple states, the question of how cross-jurisdictional service sharing among municipalities affects scope, quality and cost of FSIs, enteric disease control activities, and obesity prevention activities. The findings highlight advantages and disadvantages of two common public health service delivery models – independent and comprehensive shared services. Based on prior research studies, we hypothesized that municipalities with shared service models would be more likely to offer a greater breadth of public health services, with higher quality and at a lower cost. Our findings, discussed below, support some, but not all our hypotheses. We also found variation in some of our results by state, with some indicators more likely to be found in one state than another because of state mandates or regulations (e.g., in CT, district or shared service health departments are required to have a director with at least a MS in Public Health). By and large, however, the benefits of a two-state study outweigh these differences.

With respect to the breadth and scope of public health service delivery, we did not find significant differences between the two models in the delivery of core public health services. Services that are state mandated are most likely to be provided in sharing and independent health departments. Public health nursing was one key difference in CT only, with shared service models less likely to have public health nurses on staff than independent health departments.

Where we saw indications of difference was in non-mandated community health programs and services, such as asthma prevention, domestic violence awareness, mental health education, and obesity prevention. For example, resource-sharing departments were significantly more likely to be engaged in local healthy food and physical activity promotion activities. This aligns with other recent studies on obesity prevention activities of LHDs. A national study on factors associated with participation in obesity prevention activities using national (NACCHO) data found that health departments under 25,000 population were least likely to support obesity prevention activities (only 45%), with increases to 61% with populations of 50,000–99,999, and up to 78% for populations >500,000 (21). The same research team conducted a follow up study with semi-qualitative interviews of directors of federally funded obesity prevention programs, and found that whether a public health system was centralized (strong state lead; sharing) or decentralized (independent) was an important perceived factor in participation in obesity prevention activities, with decentralized health departments more likely to be focused on local perceived needs, and centralized health departments more likely to be concerned about lack of capacity of play a lead role (22).

During the course of answering questions about the range of public health services offered to local constituents, health directors in shared service departments noted that they were able to offer health promotion and disease prevention services because of their ability to compete for public health and social welfare grant dollars. This ability was attributed to having a diversified staff and the potential to reach a larger population than if they were to work in an independent department. Health directors from independent health departments commonly reported a lack of adequate personnel resources to provide a full range of local public health services. Those serving smaller municipalities in particular often reported feeling like a “Jack of all trades and Master of none” as they tried to meet state mandates with minimal staff. Their limited resources coupled with small population reach made it difficult to compete for grants that support prevention and intervention services. As a result, their municipalities often did not have access to the same public health services as those participating in shared service models.

In addition to breadth of services, recent studies suggest that cross-jurisdictional sharing of resources can enhance the quality of services offered (23, 24). In the present study, we found that collaborating with other municipalities provided an opportunity to create depth to their core public health service staff, allowing for certain quality measures, like supervision and evaluation, to be put into place. Although the sample size for this study was relatively small, there are indications that shared service models were able to provide at least as many mandated and more non-mandated services with the same FTE staff per 1,000 and with greater quality than independent models. The ability to provide greater breadth and quality of services with significantly fewer staff per 1,000 population is an important finding with respect to differences in efficiency between the two models.

With respect to direct costs associated with each service delivery model, we did not detect significant differences between the two models. Sharing departments have more indicators of higher quality FSIs, and more FSIs were done in sharing departments, although not on a per capita adjusted basis. Regardless of service delivery model, nearly all of the small and medium sized health departments that participated in our study did not serve populations large enough to truly see economies of scale. To achieve economies of scale in service delivery, many of the municipalities needed to share resources with a greater number of municipalities. Drawing on our findings from FSIs, for example, our data suggest that the primary driver of FSI costs is the volume of work conducted. There is a non-linear relationship between cost per inspection and number of inspections, and the minimum cost per inspection is reached above the total number of inspections conducted by all but four of the jurisdictions sampled. Service-sharing status is not significant as a factor in costs separate from the total number of inspections. However, sharing departments have more indicators of higher quality FSIs, and more FSIs were done in sharing departments, although not on a per capita adjusted basis.

Our mixed methods research design allowed us to further investigate a fuller range of factors that might influence the breadth, quality and cost of public health services than is included in most studies of local public health. One unique aspect of our study data is the inclusion of social and political indicators that were hypothesized to influence the organization and operation of local public health services. In states like MA and CT that have histories of strong home rule governance, our qualitative data highlight the importance of local authority and local values ascribed to the role of government in regulating public life. Although we found that municipalities that work in collaboration with other municipalities offer a greater breadth of services with higher quality, we also found important trade-offs and “hidden costs” of shared service models. These include the exponential amount of time and energy required to manage the expectations of multiple municipal governments and communities and to demonstrate accountability for municipal funds. This additional time and energy was often a “collateral duty” as local board of health and municipal government meetings often take place in the evenings after business hours. Participation in these meetings helped to develop constituent relations and local knowledge about the social and political differences of the municipalities they serve, but also required significant time to negotiate differences across collaborating municipalities.

Another cost that is rarely factored into studies of cross-jurisdictional service sharing is the distance that public health practitioners must travel in order to provide services to each municipality. This is particularly challenging in low density and rural areas where the distance between municipalities can be large. Health directors from shared service models talked about some of the costs associated with the distance between municipalities and the strategies they have developed to increase efficiency. We were not able to collect enough detailed information during this study to incorporate these costs and strategies into our analysis, but believe they are important to consider in future studies.

Although independent health departments were often less likely to report the same breadth of services or quality indicators as shared health departments, they report an ability to draw on strong local partnerships and intimate knowledge of their communities to meet resident needs. Frequent meetings with key representatives within their municipal government likely contributed to their belief that local governing officials had a good understanding of the roles and responsibilities of local public health. In most cases; however, this did not translate into greater allocation of local funds for public health services.

Other considerations for inclusion in future studies include examination of local governance structures. Findings from our study suggest that chief executives that are elected rather than appointed may feel more vulnerable to recurrent election cycles. Elected officials may be more reluctant to relinquish control to outside authorities through comprehensive shared service districts that would limit their authority to enact local public health ordinances or to purchase public health services from local contractors, particularly in cases where officials are satisfied they are already addressing local public health service needs.

This study adds nuance to previous work, noting that while slightly more services were provided in sharing departments, the overall differences between sharing and independent departments are small in terms of scope of services. This study also adds to limited research on effective and efficient service delivery models for small and mid-size jurisdictions, and highlights the need for more work on public health service delivery quality measures. In addition, this study extends previous research on cost of local public health services by exploring potential variations in cost by jurisdiction size and service delivery model.

### Study Limitations

There are a number of limitations to this study that are important to note. First, we recognize that CT and MA have unique local public health systems. Most local public health services in the United States are provided by county governments or through regional entities overseen by the state. However, we believe that the findings are applicable to other states, particularly those with rural counties and regions. We also think that our inclusions of political factors are of relevance to all local public health structures. With respect to data, we have some limitations to our analysis due to differences in response rates between the two states, the inability to obtain and analyze data on enteric disease infections in MA, and the inability of health directors in independent health departments to estimate overhead costs. Due to the inability to collect data on overhead costs, we had to focus all cost comparisons solely on staffing costs. In addition, municipalities engage in a wide variety of service-sharing arrangements, with no sharing and comprehensive sharing being the two extremes. To ensure comparisons were of similar models, we limited recruitment to municipalities that operated at these two extremes. As a result, we are limited in our ability to assess the impact of a full range of service-sharing models on the breadth, quality, and cost of public health service delivery.

### Implications for Practice

Although CT and MA are unique in comparison to other states with respect to the governance structure for local public health (municipality vs. county), the lessons learned through the investigation of single municipal governance structure will be applicable to single county governance structure as it applies to service-sharing arrangements. This study highlights trade-offs with each approach to public health service delivery. The size of the municipality or multiple municipalities served does matter. Independent health departments serving small jurisdictions have the most limited resources but strong local knowledge. In contrast, multi-jurisdictional models have more resources but require more time and investment in governance and decision-making. When making decisions about the right service delivery model for a given jurisdiction, careful consideration should be given to local culture and values. Investigating and identifying strategies to reconcile trade-offs between independent and shared service models offer state and local authorities the opportunity to maximize resources in promoting and protecting the health of their constituents and improving community health outcomes.

## Ethics Statement

The Yale University Human Subjects Committee and Cambridge Health Alliance Institutional Review Board reviewed this research and found it to be exempt from IRB review under federal regulation 45 CFR 46.101(b)(2).

## Author Contributions

DH, JH, EO, JK, and GW designed the study. All authors participated in the development of survey instruments, and in analysis and interpretation of the data. JH, EH, and DH were responsible for data collection. DH, JH, and EH took the lead in drafting the manuscript. All authors provided feedback on the manuscript and approved the final version.

## Conflict of Interest Statement

The authors declare that the research was conducted in the absence of any commercial or financial relationships that could be construed as a potential conflict of interest.

## Footnotes

^1^Quality indicators included: formally trained food safety inspectors; opportunities for and requirements to take part in ongoing training on food inspections; use of a standard inspection reporting form; written standard operating procedures; written policies for responding to complaints; availability of equipment needed for food inspections; annual inspection program evaluation.
