# Supplementary material for: Cross-Jurisdictional Resource Sharing in Local Health Departments: Implications for Services, Quality, and Cost
Source: Front Public Health. 2018 Apr 26;6:115. doi: 10.3389/fpubh.2018.00115 (PMC5932147; doi:10.3389/fpubh.2018.00115)
Supplement: Supplementary file 1 [file data_sheet_1.docx]

**Table S1. Measures of Food Safety, Enteric Disease and Obesity Prevention Activities**

| **Focus Area** | **Description of Measure** |
| --- | --- |
| **Food Protection** |  |
| Food safety reach | # of food service establishment inspections in past 12 months |
| Food safety volume | # of retail food inspections conducted during the past 12 months |
| Food safety field staffing ratio | # of FTE staff devoted to retail food safety inspection, protection and control per 100 retail food establishments |
| Oversight of food safety program | Resources dedicated to supervision and oversight of inspectional service work |
| Staffing cost for food safety program | Amount of money it costs per unit of service delivery, including travel, documentation, and supervision |
| **Foodborne/Waterborne Enteric Disease** | |
| Reported case volume | # of reported cases of enteric disease in past 12 months. |
| Confirmed case volume | # of confirmed cases of enteric disease in the past 12 months |
| Investigation volume | # of investigations of reported foodborne/enteric disease cases conducted by LHD during the past 12 months |
| Staff cost to perform investigation | Amount of money it costs per unit of service delivery, including staff costs |
| **Obesity Prevention** | |
| Availability of physical activity interventions | Availability community-based physical activity interventions, resources to increase physical activity & reduce obesity risk |
| Availability of healthy food/eating interventions | Availability of community-based healthy food interventions, resources to increase healthy eating practices and reduce obesity risk |
| LHD staff contributions to physical activity interventions | Cost of total staff FTE allocated to physical activity interventions |
| LHD staff contributions to healthy food initiatives | Cost of total staff FTE allocated to health food access in the community |

**Table S2: Physical Activity Initiative Questions**

|  | **What role did the LHD play in this activity?** | | | | |
| --- | --- | --- | --- | --- | --- |
| **Which of the following community-wide physical activity interventions have been underway within your jurisdiction(s) during the past 12 months? *[If interviewing a district, write the number of municipalities for which the option is appropriate.]*** | **No role** | **Minor collaborating partner** | **Major collaborating partner** | **Co-lead** | **Lead** |
| Community-wide health education campaigns (e.g., large-scale, highly visible, messages directed to broad audiences through media such as television, radio, and newspapers, typically combined with other approaches including support or self-help groups, community events, or risk factor screenings). |  |  |  |  |  |
| Community-wide stair use campaigns (e.g. motivational signs placed by elevators/escalators to encourage people to use nearby stairs for health/weight loss). |  |  |  |  |  |
| School-based PE programs (e.g. programs to increase amount of time students spend in PE classes which enhance the length or activity level of students and health education). |  |  |  |  |  |
| Social support interventions in community (e.g. focus on changing physical activity behavior through creating, strengthening and maintaining social networks that provide supportive relationships for behavior change). |  |  |  |  |  |
| Individually adapted health behavior change programs (e.g. teaching goal setting/self monitoring of progress, structured problem solving and relapse prevention). |  |  |  |  |  |
| Initiatives to create or enhance access to places for physical activity combined with informational outreach activities e.g. (built environment walking trails, biking trails, exercise facilities within worksites/coalitions, agencies). |  |  |  |  |  |
| Community-level urban design initiatives (e.g. developments to increase the % of residents living within walking distance of shopping, work, & school, improved connectivity of streets and sidewalks, preserve or create green-space & improve aesthetic qualities of the environment). |  |  |  |  |  |

**Table S3. Healthy Food Initiative Questions**

|  | **What role did the LHD play in this activity?** | | | | |
| --- | --- | --- | --- | --- | --- |
| **Was your LHD involved in any initiatives to increase access to healthy foods in the community in the past 12 months? If yes, what type of initiatives was the LHD involved in? [If interviewing a district, write the number of municipalities for which the option is appropriate.]** | **No role** | **Minor collaborating partner** | **Major collaborating partner** | **Co-lead** | **Lead** |
| Initiatives to change the foods/beverages available at schools and school-sponsored events. |  |  |  |  |  |
| Initiatives to change the foods/beverages served and/or sold by government agencies and government-sponsored programs |  |  |  |  |  |
| Initiatives to change the foods/beverages served and/or sold by restaurants, food service establishments, and food vendors in the community |  |  |  |  |  |
| Policies to require menu labeling and calorie disclosure for the foods/beverages served and/or sold by restaurants, food service establishments, and food vendors in the community |  |  |  |  |  |
| Initiatives to promote the availability of grocery stores, farmer’s markets, and other fresh produce outlets in the community |  |  |  |  |  |
| Initiatives to promote the availability and/or use of community gardens |  |  |  |  |  |
| Initiatives to change the price of foods/beverages in school and/or community settings |  |  |  |  |  |
| Initiatives to change the marketing practices used for foods/beverages in school and/or community settings |  |  |  |  |  |
| Initiatives to promote and facilitate breastfeeding in clinical, workplace, and community settings. |  |  |  |  |  |
| Other initiatives by your agency to increase access to healthy foods in the community. Please specify: |  |  |  |  |  |
